# Assessment of Susceptibility to Five Common Antibiotics and Their Resistance Pattern in Clinical Enterococcus Isolates

**DOI:** 10.30699/IJP.2020.114009.2236

**Published:** 2020-02-19

**Authors:** Sara Masoumi Zavaryani, Reza Mirnejad, Vahhab Piranfar, Mehrdad Moosazadeh Moghaddam, Nikta Sajjadi, Somayyeh Saeedi

**Affiliations:** 1 *Department of Microbiology, Islamic Azad University of Varamin-Pishva Branch, Tehran, Iran *; 2 *Molecular Biology Research Center, Systems Biology and Poisonings Institute, Baqiyatallah University of Medical Sciences, Tehran, Iran*; 3 *Research and Development Department, Farname Inc., Thornhill, Canada*; 4 *Applied Biotechnology Research Center, Baqiyatallah University of Medical Sciences, Tehran, Iran*; 5 *CNC, Center of Neuroscience and Cell Biology, University of Coimbra, Coimbra, Portugal*; 6 *Department of Microbiology, Faculty of Advanced Sciences and Technology, Pharmaceutical Sciences Branch Islamic Azad University, Tehran, Iran (IAUPS)*

**Keywords:** Enterococcus faecalis, Enterococcus faecium, Multiple drug resistance, Correlation

## Abstract

**Background & Objective:**

Enterococcus Species are the common cause of nosocomial infections, which are highly resistant to different antibiotics. Therefore, determination of their antibiotic susceptibility patterns and simultaneous resistance to antibiotics is important for better treatment strategies.

**Methods:**

400 clinical *Enterococcus *isolates were collected from different hospitals in Tehran, Iran. Standard phenotypic-biochemical tests and PCR were used to identify the *Enterococcus *species. The antimicrobial susceptibility patterns and simultaneous resistance to selected antibiotics were determined by disk diffusion method according to the CLSI guidelines. All data analysis was performed using Python packages Scipy and Stats models.

**Results:**

According to the biochemical and PCR analyses, among 400 *Enterococcus *species, 72% of samples were *Enterococcus faecalis*, 10.75% *Enterococcus faecium*, and 17.25% other *Enterococcus *species. The results determined antimicrobial resistances of these strains against gentamicin, vancomycin, fosfomycin trometamol, teicoplanin, and quinupristin/dalfopristin. Results confirmed a significant correlation between resistance to vancomycin and resistance to teicoplanin. This correlation remains significant when including only *E. faecium* or *E. faecalis* species. We also found a negative correlation between resistance to teicoplanin and quinupristin/dalfopristin. Additionally, Quinupristin/dalfopristin was the least effective antibiotic while vancomycin and teicoplanin were the most effective ones.

**Conclusion:**

Based on the results and association between simultaneous resistance to some antibiotics such as vancomycin and teicoplanin, in the case of antibiotic resistance, the choice of a second antibiotic can be very important which can lead to good or bad effects.

## Introduction

*Enterococcus *species are a major part of the gastrointestinal tract which is responsible for 10% of hospital-acquired infections (1-3). The most common human infectious strains of *Enterococcus* are *E. faecalis* (85–90%) and *E. faecium* (10-15%) leading to urinary tract infections, endocarditis, bacteremia, wound infection, abdominal infections, pelvic infections, and meningitis (4). On the other hand, about 30% of all nosocomial bloodstream infections are associated with *Enterococcus *species and *Staphylococcus aureus*, resulting in significant morbidity and mortality (5-8). Based on the United States Nosocomial Infections Surveillance System’s data, *Enterococci* are considered as one of the nosocomial pathogens (9). These bacteria are ranked fourth in nosocomial infectious agents, third in bacterial infections, and second in pathogens causing urinary tract infections, which has prompted some to consider a worldwide emergence of antibiotic-resistance in these species (10). Since these bacteria can live in a wide range of environments, their identification is essential for controlling and prevention of infections (11-13). On the other hand, *Enterococci* are tolerant to the bactericidal activity of cell-wall active agents, such as β–lactam antibiotics and vancomycin. *Enterococcal* tolerance to these antibiotics can be affected by combining cell-wall active agents with an aminoglycoside based on synergistic bactericidal activity. Studies have shown that a higher concentration of aminoglycoside enters cells that are also treated with agents that inhibit cell wall synthesis, which suggests that the cell wall active agents promote uptake of the aminoglycoside (5, 14). Accordingly, to treat infections caused by *Enterococci*, combination therapy with a cell wall–active agent and a synergistic aminoglycoside should be considered. Nevertheless, in recent years, resistance to aminoglycosides and decreased susceptibility to β-lactam antibiotics and vancomycin, makes their synergistic function less efficient (15-19). Therefore, the widespread resistance of *enterococci* has a significant impact on the selection and use of synergistic antibiotics for the treatment of *enterococcal* infections. Given the importance of this issue, in this study, we collected clinical samples to contain different *Enterococcus* species and then analyzed resistance pattern of each sample against five common antibiotics. In the following, the correlation between resistance to antibiotics and simultaneous resistance to selected antibiotics was investigated. The findings can help better understand the trends of antibiotic resistance of Enterococcus species, and guide strategies for the use of antibiotics.

## Materials and Methods

**Sample Collection**

We conducted a cross-sectional study on 400 clinically *Enterococcus *spp. Samples (urine, wound, blood, ascites, etc.) were randomly collected from Baqiyatallah and Milad hospitals (Tehran, Iran), from January to December 2017. The samples were collected from patients of all age groups and both genders, without any restrictions on the cause of hospitalization.

**Identification of Enterococcus Species**

**-Phenotypic-Biochemical Tests**

To identify *Enterococcus* species by biochemical test, 24-hour pure blood agar medium was produced. Next, the following tests were performed on each sample: gram staining, catalase test, bile salt hydrolysis (40% bile salts), growth on Brain Heart Infusion (BHI) medium containing 6.5% salt (NaCl), and sugar fermentation tests of arabinose, mannitol, sorbitol, sorbose and lactose (20).

***-PCR Analysis***

For identification by PCR, the DNA of *Enterococcus* species was extracted using the boiling method (21). Commercially synthesized primers specific to genes (D-AlaD-Ala) of *E. faecalis* and *E. faecium* were obtained from Pishgam Biotech Company (Tehran, Iran) (21-23μμμμμμ*E. faecalis *and 550 bp for *E. faecium*) were analyzed by electrophoresis using 1.5% agarose gel and visualized and analyzed by Safe Satin staining with the help of Gel Documentation system (Cambridge, England, Uvitec) and a 100 bp DNA Ladder (Green BioResearch LLC, USA). The amplified PCR products were confirmed by sending the samples for sequencing (Bioneer, Korea).

**Table 1:** The sequence of the primers used in PCR amplification of (D-Ala D-Ala) *E. faecalis* and (D-Ala D-Ala) *E. faecium* genes

Target genes	Primer sequence (5’ → 3’)	Amplicon size	Reference
**(D-Ala D-Ala)** ***E. faecalis***	Forward: ATCAAGTACAGTTAGTCT Reverse: ACGATTCAAAGCTAACTG	941 bp	(56)
**(D-Ala D-Ala)** ***E. faecium***	Forward: TAGAGACATTGAATATGCC Reverse: CTAACATCGTGTAAGCT	550 bp	(56)

**Antimicrobial Susceptibility Tests**

Susceptibility tests for antibiotics (Mast Group, Merseyside, UK) including gentamicin (10 µg), vancomycin (30 µg), teicoplanin (30 µg), fosfomycin trometamol (50 μg) and quinupristin/dalfopristin (15 µg) were performed on Mueller-Hinton agar (Merck Co., Germany) plates using disc diffusion method according to the guidelines of Clinical and Laboratory Standards Institute (CLSI) (24). *E. faecalis *ATCC 2921 (25) was used as a reference strain for antibiotic susceptibility tests. Also, according to the CLSI recommendation for *Enterococcus* species, minimum inhibitory concentration (MICs) of vancomycin was performed for resistant isolates by microdilution method in BHI broth medium and different concentrations (4 – 1024 μg/mL) of antibiotic (24, 26).

**Statistical Analysis**

We performed all data analysis using Python packages Scipy (version 0.19.1) and Stats models (version 0.8.0).

## Results

**Sample Distribution**

Of 400 *Enterococcus *isolated clinical samples, 83.75% (335 samples), 6% (24 samples), and 3.25% (13 samples) were isolated from urine, wound, and blood, respectively and 7% (28 samples) were isolated from other locations (vagina, sputum, ascites, and Bronchoalveolar lavage). All age groups entered the study (maximum age of 87 years). There were 185 (46.25%) males and 215 (53.75%) females.

**Identification of Enterococcus Species**

**Fig. 1:**
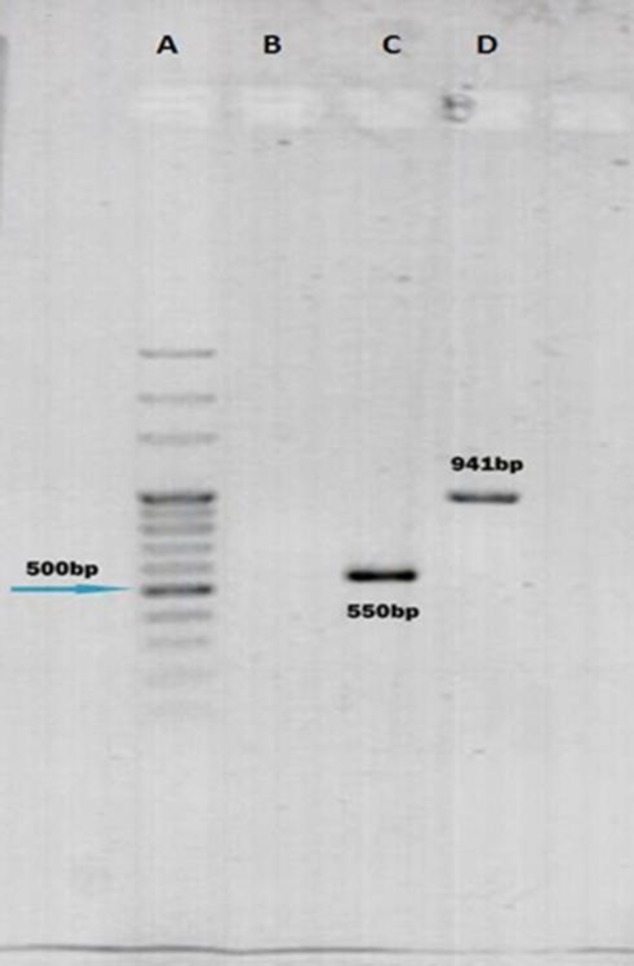
An example of gel electrophoresis of PCR products used to identify *Enterococcus* species. Lane A. is marker DNA (100 bp), Lane B. is non-template DNA sample, Lane C. is an amplified (D-Ala D-Ala) *E. faecium* (550 bp) gene in clinical samples examined, Lane D. is an amplified (D-Ala D-Ala) *E. faecalis* (941 bp) product of clinical samples examined

PCR results showed that 288 (72%) isolates were of *E. faecalis*, 43 (10.75%) *E. faecium *and the remaining 69 (17.25%) other *Enterococcus* species. PCR was mainly used to identify *E. faecalis* and *E. faecium* (Figure 1). Using a BHI+ NaCl 6.5% test (27), we confirmed that these 69 strains were from other *Enterococcus* species.

**Antibiotics Resistance Pattern & Association Between Simultaneous Resistance to Selected Antibiotics**

**Fig. 2:**
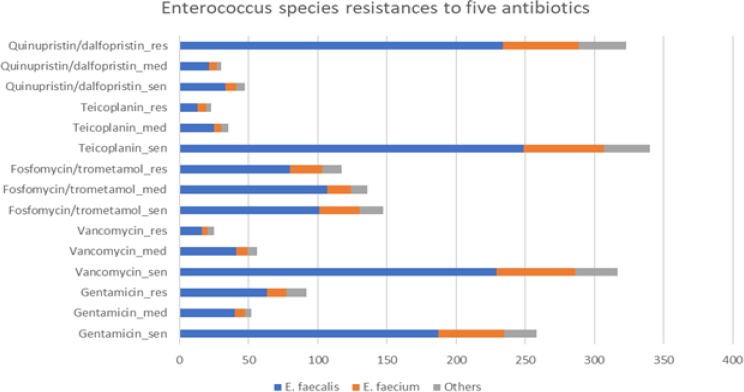
*Enterococcus* species and their resistance to five different antibiotics. A suffix of "sen" means sensitive to that antibiotic, "med" mean intermediate resistance, and "res" means resistant

Kirby-Bauer antibiotic tests (28) were performed to identify *Enterococcus* isolates resistant to Gentamicin, vancomycin, teicoplanin, fosfomycin trometamol, and quinupristin/dalfopristin. Antibiotic resistance patterns in bacteria samples are shown in Figure 2. Isolated samples were categorized based on their origin, i.e., urine, blood, and wound samples, or samples from sites that we labeled as “others”, due to their low frequencies. The “other” sites from which samples were taken include the vagina, sputum, ascites, and bronchoalveolar lavage. Samples categorized as sensitive, semi-sensitive or resistant to each antibiotic using disk diffusion method, according to the guidelines of CLSI (24).

Our results confirmed that resistance to teicoplanin is correlated with resistance to quinupristin/dalfopristin and vancomycin. Accordingly, we found a strong correlation between the resistance of samples to vancomycin and teicoplanin (Pearson’s r=0.36, *P*=8.44×10^-14^). The two antibiotics also showed significant correlations when we included only *E. faecalis* (Pearson’s r=0.36, *P*= 3.71×10^-10^) or *E. faecium* (Pearson’s r=0.63, *P*=5.21 ×10^-6^) species. Indeed, the correlation was considerably stronger when considering only *E. faecium*. Furthermore, there was a nearly significant and negative correlation between resistance to quinupristin/dalfopristin and teicoplanin (Pearson’s r= -0.10, *P*= 0.05). This correlation was very significant, if we consider non-sensitivity of samples to the antibiotics, that is, samples that are completely or partially resistant to the two antibiotics (Pearson’s r= -0.15, *P*= 2.44 ×10^-3^). This correlation becomes stronger only within the other *Enterococcus* species (Pearson’s r= -0.34, *P*= 4.64 ×10^-3^), but within *E. faecium* or *E. faecalis*, there is no significant correlation. On the other hand, the *Enterococcus* species are most resistant to quinupristin/dalfopristin (323 samples). This fraction is significantly more than the resistance to any other antibiotic (Fisher’s exact test, corrected for multiple testing by using false discovery rate (FDR) (Tables 2 and 3) (29). After that, and by a large distance, the least effective antibiotics are fosfomycin/trometamol and gentamicin with 117 and 90 resistant samples, respectively. They are both significantly less effective than a teicoplanin and vancomycin (Table 2). There was no significant dif-ference in the effectiveness of fosfomycin/trometamol and gentamicin. The most effective antibiotics were teicoplanin and vancomycin, with only 23 and 27 samples were resistant to them, respectively (Table 3). 

GM: gentamicin, VAN: vancomycin, FOT: fosfomycin trometamol, TEC: teicoplanin, SYN: quinupristin/dalfopristin.

**Table 2:** Correlation between resistance to selected antibiotics in isolated samples. There are significant differences between the numbers of resistant samples to one antibiotic versus the other. The rows are Fisher’s exact test’s odds ratio and its p-value, corrected for multiple testing by using FDR. The columns are comparisons between pairs of antibiotics

Antibiotics										
	GM-VAN	GM-FOT	GM-TEC	GM-SYN	VAN-FOT	VAN-TEC	VAN-SYN	FOT-TEC	FOT-SYN	TEC-SYN
**odds ratio**	0.24933	1.42403	0.210139	14.4488	5.71143	0.842814	57.9505	0.147566	10.1464	68.7583
**p-value**	2.88E-10	0.039638	8.26E-12	7.21E-64	5.27E-17	0.661648	1.19E-110	5.92E-19	4.31E-50	1.43E-114

GM: gentamicin, VAN: vancomycin, FOT: fosfomycin trometamol, TEC: teicoplanin, SYN: quinupristin/dalfopristin.

**Table 3:** The number of resistant samples to each antibiotic

Antibiotic	Number of resistant samples
***GM***	90
***VAN***	27
***FOT***	117
***TEC***	23
***SYN***	323

There was no difference between resistance to different antibiotics in *E. faecium* and *E. faecalis* (Fisher’s exact test, Table 4). According to the results, a large fraction of samples was resistant to multiple antibiotics. A minimum of 42.4% (*E. faecalis*) and a maximum of 58.1% (*E. faecium*) of samples were resistant to more than one antibiotic (Table 5). Most multi-resistant species were resistant to only two antibiotics, but between 1-2% of the samples were resistant to four antibiotics at the same time (Table 5). There was, however, no difference between the fractions of samples that were multi-resistant in different species. The number of samples that were co-resistant to each pair of the antibiotics is shown in Table 6. Co-resistance occurs between all pairs of antibiotics. The most common co-resistance occurred in case of fosfomycin/trometamol and quinupristin/dalfopristin (100 samples), and between gentamicin and quinupristin/dalfopristin (76 samples). The least common co-resistance was between gentamicin and vancomycin (6 samples). According to the findings, age and sex had no effect on resistance to any antibiotic. Using generalized linear models with logistic regression, we found no effect of age, sex or their combination on resistance to antibiotics.

**Table 4:** The number of resistant samples (*E. faecalis* and *E. faecium*) to different antibiotics. The last column shows Fisher’s exact test p-values (corrected for multiple testing using FDR) for any difference between the numbers of resistant samples of the two species

Antibiotics	E. faecalis	E. faecium	P-value
***GM***	61	15	0.27
***VAN***	18	5	0.42
***FOT***	80	14	0.68
***TEC***	13	4	0.42
***SYN***	234	34	0.68

**Table 5:** The fraction of samples that are simultaneously resistant to two or more antibiotics. The first column shows the number of antibiotics to which there is simultaneous resistance, and other columns show the fraction of all samples or fraction of samples within different species which are resistant to multiple antibiotics simultaneously. The last row is the sum of all rows above it

Simultaneous resistance (Number of antibiotics )	All species	E. faecalis	E. faecium	Other species
**2**	0.343	0.330	0.419	0.348
**3**	0.090	0.080	0.140	0.101
**4**	0.015	0.014	0.023	0.014
**5**	0	0	0	0
sum	**0.448**	**0.424**	**0.581**	**0.464**

**Table 6:** The number of samples with co-resistance to different antibiotics by the antibiotics

Samples	GM-VAN	GM-FOT	GM-TEC	GM-SYN	VAN-FOT	VAN-TEC	VAN-SYN	FOT-TEC	FOT-SYN	TEC-SYN
***All species***	6	25	7	76	11	10	23	8	100	15
***E. faecalis***	2	16	3	53	8	6	17	5	69	9
***E. faecium***	2	3	2	12	1	3	3	1	13	2
**Other species**	2	6	2	11	2	1	3	2	18	4

## Discussion

 Over the past two decades, due to excessive consumption of antibiotics, resistance to common antibiotics has been increased (30-32). Accordingly, infections with methicillin-resistant *S. aureus *(MRSA) and vancomycin-resistant *Enterococcus* species (VRE) poses significant treatment challenges, which leads to an increase in treatment failure, relapse, and higher rates of mortality, as according to the reports mortality from enterococcal bacteremia is estimated at 15–35% (33). Vancomycin resistance in *Enterococcus *species has been increased in hospitalized patients and affected the treatment of *Enterococcus* infections (34-38). A report by the National Healthcare Safety Network in the United States shows that approximately 40% of majority of device-associated infections, such as urinary drainage catheters and ventilators, are associated with vancomycin- and ampicillin-resistant *E. faecium* with a prevalence of 80% and 90.4%, respectively. While, infections in these units which caused by *E. faecalis* remained largely susceptible to ampicillin and vancomycin (96.2% and 93.1%, respectively) for reasons that are not entirely known (16, 39). However, molecular analyses have shown that *E. faecium *is intrinsically more resistant to antibiotics than *E. faecalis*, so that more than a half of the pathogenic isolates of this bacterium show resistance to vancomycin, ampicillin, and high-levels of aminoglycosides (40,41). According to clinical studies, many hospital-associated strains that are resistant to vancomycin also show resistance to penicillin, as well as high-level resistance to aminoglycosides. Therefore, the specific and accurate identification and determination of *Enterococcus *species and their antibiotic resistance pattern is important to provide an effective treatment protocol and the choice of right drug to treat infection and to avoid transfer of vancomycin-resistant plasmid from *Enterococcus* to main pathogen bacteria and other *Enterococcus* strains (42,43). Combination antibiotic therapy can be a significant strategy for treating infections caused by *Enterococcus *species. Data showing that this strategy can lead to improved rates of cure and lower rates of relapse when compared to monotherapy (5,18). Currently, combination therapy of a cell wall-active agent such as vancomycin, teicoplanin, and fosfomycin trometamol plus an aminoglycoside like gentamycin and quinupristin/dalfopristin is as a standard protocol for treatment of enterococcal infections (44). It has been shown that the use of aminoglycosides with penicillin as cell wall-active antibiotic produced synergistic activity and improve the cure rates for enterococcal infective endocarditis from 40 to 88% (45). However, although the recommended regimens currently include the use of two or more antibiotics, inappropriate and long-term use of these antibiotics can also lead to drug resistance (30,44). Therefore, the correct selection of antibiotics and understanding the relationship between antibiotic resistances can reduce this risk. Accordingly, in the current study, 72% of samples were infected with *E. faecalis*, 10.75% with *E. faecium*, and 17.25% with other *Enterococcus* species. Our results showed that among *E. faecalis* and *E. faecium* isolates, resistance to cell wall-active antibiotics (vancomycin, teicoplanin, and fosfomycin trometamol) were 33.5% and 53.5%, respectively, which are consistent with its global prevalence (8, 40, 46). However, among *E. faecalis* and *E. faecium* isolates the highest resistance was to fosfomycin trometamol antibiotic (27% and 32%, respectively) while for the other two antibiotics it was almost the same (5.5% and 10%, respectively). In this study, a high rate of resistance to fosfomycin was observed, while this antibiotic is as an alternative antibiotic against multidrug resistant organisms, including vancomycin-resistant enterococcus (VRE) and extended-spectrum β-lactamase (ESBL) (47) which could be due to its excessive and inappropriate use. In addition, according to many reported studies (48-50), *E. faecalis* and *E. faecium* isolates exhibit high resistance to aminoglycosides including gentamycin and quinupristin/dalfopristin. Our findings showed that 21% of *E. faecalis* and 35% of *E. faecium* isolates are resistant to gentamycin while a high number of isolates were resistant to quinupristin/dalfopristin (81% and 79%, respectively).

Similar to this study, there have been extensive studies in Iran and other countries. For instance, in a study by Shahraki *et al.*, (2017) 182 samples were collected from southeast of Iran. Among samples, 63 and 22 cases were caused by *E. faecalis* and *E. faecium* strains, respectively. According to their reports, only 6 *E. faecalis* and 12 *E. faecium* isolates were resistant to vancomycin (51), which is different from our results because more than 50% of *E. faecium* show resistance to vancomycin while in current study the resistance rate is about 10%. In addition, Arbabi *et al.*, (2016) determined 149 *Enterococcus* species and their resistance pattern isolated from clinical samples of some hospitals in Tehran, Iran. Among isolates, 60% and 26% were of *E. faecalis* and *E. faecium*, respectively. About 33 strains of VRE, more than a half of the isolates were *E. faecium*, and *E. faecalis* was in the second place (52). In contrast to the findings in these studies, a lower prevalence of *E. faecalis *has been reported by Labib Azza *et al.*, (2013) in Egypt (53). They identified *Enterococcus *species by phenotypic and molecular methods and found significant differences between the frequency of *E. faecalis *(32%) and *E. faecium *(48%) infections. In addition, 60% isolates were identified as VRE. Also, a study in Iraq by Al-Hadithi and Rasheed (2018) (54) showed that among 57 isolates of *E. faecalis* (N=42) and *E. faecium* (N=15) which were isolated from infected wounds higher percentage of vancomycin resistance is associated with *E. faecium* (53.3%) as compared to *E. faecalis* (47.6%), which is similar to other studies performed in Iran. It is noteworthy that compared to these studies, our results showed that more than 90% of the isolated samples were susceptible to vancomycin.

Generally, in the present study, we found resistance to the first-line treatment, i.e., aminoglycosides. We also found resistance to substituting antibiotics such as vancomycin and teicoplanin, although at lower levels especially for vancomycin. However, according to the studies the high transformability of glycopeptides in *Enterococci* help develop resistance to different antibiotics (55). On the other hand, the statistical analysis of resistance in the *Enterococcus *species showed a prevalence of multi-resistant species. More than 40% of samples from different species are resistant to more than two antibiotics, and a small fraction of 1%-2% have gained resistance to four antibiotics (Table 5). This can be an alarming beginning of increased resistance to common antibiotics in *Enterococci*, especially that there are no two antibiotics in our list to which co-resistance has not evolved. We found a strong positive correlation between resistance to vancomycin and teicoplanin with similar rate of resistance. This suggests that if one of these two antibiotics was not effective in the treatment of an *Enterococci* infection, the other one will likely not be effective and should not be prescribed, because, the mechanism of action of vancomycin and teicoplanin, both from glycopeptides family, is the same (55). These inhibit growth of bacteria by interfering peptidoglycan biosynthesis. Additionally, we found a negative correlation between sensitivity to vancomycin and fosfomycin trometamol. Fosfomycin trometamol, a broad-spectrum penicillin, despite having a similar mechanism of action to teicoplanin, is effective on strains with resistance to vancomycin and vice versa. The effectiveness of different of antibiotics with respect to one another is shown in Tables 2 and 3. Briefly, teicoplanin and vancomycin are the most effective antibiotics, followed by fosfomycin trometamol and gentamicin. quinupristin/dalfopristin, being ineffective on 80.75% (323) of the samples, seems to be a poor choice to treatment of *Enterococci* infections. 

## Conclusion

In sum, we showed a detailed resistance pattern of clinically isolated *Enterococci* species to five common antibiotics. Our results are not merely descriptive; using statistical analysis, we distinguish between resistance patterns that may have occurred due to chance alone and patterns that are unlikely to have occurred by chance. Our findings showed positive and negative correlations between the resistance to common antibiotics in these bacteria. Accordingly, the results confirmed the association between simultaneous resistance to vancomycin and teicoplanin. These results can guide antibiotic prescriptions against *Enterococci* infections.
